# The urgent need for more potent antiretroviral therapy in low-income countries to achieve UNAIDS 90-90-90 and complete eradication of AIDS by 2030

**DOI:** 10.1186/s40249-019-0573-1

**Published:** 2019-08-02

**Authors:** Emmanuel Ndashimye, Eric J. Arts

**Affiliations:** 1 Department of Microbiology and Immunology, Western University, 1151 Richmond St., DSB Rm.3007, London, ON N6A5C1 Canada; 2 Center for AIDS Research Uganda Laboratories, Joint Clinical Research Centre, Kampala, Uganda

**Keywords:** HIV-1 drug resistance, Integrase inhibitors, Antiretroviral therapy, Low-income countries, Adherence, UNAIDS 90–90-90 target, AIDS free generation

## Abstract

**Background:**

Over 90% of Human Immunodeficiency Virus (HIV) infected individuals will be on treatment by 2020 under UNAIDS 90–90-90 global targets. Under World Health Organisation (WHO) “Treat All” approach, this number will be approximately 36.4 million people with over 98% in low-income countries (LICs).

**Main body:**

Pretreatment drug resistance (PDR) largely driven by frequently use of non-nucleoside reverse transcriptase inhibitors (NNRTIs), efavirenz and nevirapine, has been increasing with roll-out of combined antiretroviral therapy (cART) with 29% annual increase in some LICs countries. PDR has exceeded 10% in most LICs which warrants change of first line regimen to more robust classes under WHO recommendations. If no change in regimens is enforced in LICs, it’s estimated that over 16% of total deaths, 9% of new infections, and 8% of total cART costs will be contributed by HIV drug resistance by 2030. Less than optimal adherence, and adverse side effects associated with currently available drug regimens, all pose a great threat to achievement of 90% viral suppression and elimination of AIDS as a public health threat by 2030. This calls for urgent introduction of policies that advocate for voluntary and compulsory drug licensing of new more potent drugs which should also emphasize universal access of these drugs to all individuals worldwide.

**Conclusions:**

The achievement of United Nations Programme on HIV and AIDS 2020 and 2030 targets in LICs depends on access to active cART with higher genetic barrier to drug resistance, better safety, and tolerability profiles. It’s also imperative to strengthen quality service delivery in terms of retention of patients to treatment, support for adherence to cART, patient follow up and adequate drug stocks to help achieve a free AIDS generation.

**Electronic supplementary material:**

The online version of this article (10.1186/s40249-019-0573-1) contains supplementary material, which is available to authorized users.

## Multilingual abstracts

Please see Additional file 1 for translations of the abstract into the five official working languages of the United Nations.

## Background

In 2014, the Joint United Nations Programme on HIV/AIDS (UNAIDS) and partners launched the 90–90-90targets. The 90–90-90 global targets by UNAIDS call for 90% of all people living with human immunodeficiency virus (HIV) to know their status, 90% of all people diagnosed with HIV infection to be on combined antiretroviral therapy (cART) and 90% of all people receiving cART to have suppressed the virus by 2020 [1]. The world is progressing towards achieving these targets with most European countries close to 90–90-90 targets [2]. Some countries like Denmark, Iceland, Sweden, Singapore, and the United Kingdom have already achieved these targets. Good progress has been realized in Eastern and Southern Africa, and countries in West and Central Africa are lagging far behind. There has also been least progress realised in Eastern Europe and central Asia [3]. In a recent report by UNAIDS, antiretroviral therapy (ART) was accessible to only 26% of children and 41% adults in Western and Central Africa, compared to 59% of children and 66% adults who had access to ART in Eastern and Southern Africa in 2017. There was also almost 50% less reduction in number of AIDS related deaths in West and Central Africa compared to Eastern and Southern Africa (24% vs 42% respectively) [4]. LICs carry 90% of global HIV burden, and though there has been good progress to achieve 90–90-90 targets, pretreatment drug resistance (PDR), non-adherence, and side effects associated with the current cART pose a great threat. PDR and transmitted drug resistance (TDR) are on rise in low-income countries (LICs) [5, 6] and the trend is not likely to change as countries implement World Health Organisation (WHO) “Treat All” recommendation [7]. It has been shown that increase of TDR results in increased treatment switches [8] and positively correlates with cART roll out. However, with limited treatment options, these countries are facing a dilemma of keeping patients on variable regimen. Lack of access to drugs with high genetic barrier to resistance in this setting contributes to the transmission of HIV resistant virus, and limited virological monitoring affects early detection of drug resistant mutations (DRMs). In LICs, first line (FL) cART commonly consists of two nucleoside reverse transcriptase inhibitors (NRTIs) and one non-nucleoside reverse transcriptase inhibitors (NNRTIs) commonly efavirenz (EFV) or nevirapine (NVP), with Lopinavir/Ritonavir (LPV/r) or Atazanavir/Ritonavir (ATV/r) replacing NNRTI in second line (SL) therapy. More potent drugs, Darunavir (DRV), Raltegravir (RAL), and Etravirine (ETR) are sparingly used and commonly assessed by patients on salvage therapy and only in a few treatment centres.

Routine viral load monitoring, more potent drugs with fixed dose combinations with higher genetic barrier to DR and adherence support are among areas of emphasis for rapid scale up and better care of patients [9]. However, these guidelines have hardly been implemented in LICs due to lack of access to such drugs. It has been predicted that with no change of current regimens and TDR increasing beyond 10%, there will be 890 000 new deaths, 450 000 new infections and increase of cART cost of USD 6.5 billion by 2030 [10]. This commentary discusses cART-based reasons underlying these challenges and mitigation strategies.

## Main body

Over the last two decades, access to ART by HIV infected patients in countries most hit by HIV epidemic has increased. Contrary to high income countries (HICs), most HIV patients in LICs have access to the type of ART with high pill burden, side effects, and easily counteracted by resistance. These have translated into frequent drug switches which are not sustainable, and therefore likely to have a huge impact to achieving 90–90-90 goal and ultimate target of eradicating HIV by 2030.

### The low genetic barrier to drug resistance

The commonly used NNRTIs in FL, (NVP/EFV), and (LPV/r) based SL therapy in LICs, have low genetic barrier to drug resistance compared to second generation NNRTIs, rilpivirine (RPV) and ETR. High-level resistance between these drugs has been reported [11] which reduces treatment response more so in patients with TDR. In Uganda for example, where NFVand EFV are frequently used in first line, over 96% FL failures, 75% SL failures, and 49% RAL failures, have NNRTIs resistance [12]. In LICs, over two-thirds of patients retain NNRTIs resistance even many years on second line therapy [12], and Steegen et al. show that ≥65% of second line failures had NNRTI mutation [13]. Lack of HIV genotypic tests in most countries impedes early detection of drug resistant variants. Already HIV drug resistance (HIVDR) in patients failing LPV/r-based regimen has increased in Africa [14], and the number of patients who need second line therapy is likely to increase from 0.5–3.0 million in 2020 to 0.8–4.6 million by 2030. From 2012, Tenofovir (TDF) is the primary NRTI for first-line cART after replacing zidovudine and Stavudine. However, TDF resistance is on rise in LICs with 60% of patients who fail on TDF based cART having TDF resistance [15] and this could be due to lack of baseline resistance testing and prescription of TDF with EFV or NVP which have low genetic barrier to resistance.

### Side effects of current regimens and poor adherence

Adherence to treatment is critical to a successful treatment response and may be influenced by cART. It reverses occurrence of mortality, cART related morbidity, hospital visits, and improves immunological benefit of using ART. Improved adherence correlates with increased CD4 count [16] and is the second-best predictor of disease progression [17]. Poor adherence may be associated with development of DRMs which may contribute to virological failure (VF). Studies have shown that contrary to the belief that people in LICs are naturally non-adherent to treatment, adherence can also be achieved in LICs [18].

The available cART in LICs is complex and associated with huge pill burden, short, and long-term medication side effects. Adherence was reported to be around 40% in a recent adolescents study in 23 sub-Saharan Africa countries [19]. The study shows non-adherence as a key problem facing health care service in this region, and it is worsened by counselling services focusing on outcomes of non-adherence not causes. Short message service and treatment supporters can improve adherence in Africa [20], however, such strategies should go in hand with availability of more potent drugs with better safety and tolerability profiles.

Though LPV/r is associated with more adverse gastrointestinal effects than ATV/r or darunavir/ritonavir [21], it still forms the backbone of majority of second line treatment. It has recently been shown to increase cardiovascular risks of myocardial infarction and stroke in HIV infected patients in the US when compared to ATV/r [22]. Boosted lopinavir and 2NRTI combination has been removed from other regimens category because of huge pill burden and greater toxicity (//aidsinfo.nih.gov/guidelines). HIVDR resulting in VF in second line failures in LICs, has been attributed to poor adherence other than LPV/r activity [23]. In addition, the commonly used NNRTI, EFV, is associated with more adverse gastrointestinal effects and rashes compared to RPV [24].

### Pretreatment HIV-1 drug resistance

Globally, over 10.1% of HIV infected patients have baseline drug resistance [25] and it is associated with reduced treatment response in both HICs [26] and in LICs [27]. Whereas prevalence of TDR remained stable in HICs 2002–2010 at 8%, in LICs, there has been an increase in prevalence with roll out of cART. In some countries, frequencies of NNRTI and NRTI DRMs in patients initiating cART increased from 0% (2006–2007) [28], to 8.6% (2009–2010) [29] and 15.4% (2014–2016) [6]. Despite an estimated prevalence of HIVDR of (7.4%) eight years after roll-out of cART, the estimated annual increase of PDR in East Africa is 29 and 14% in South Africa and largely driven by high NNRTIs resistance [6]. This confirms observed positive correlation between cART roll out and increase of TDR in LICs [30]. In a national survey by 11 LICs countries on PDR, six out of 11 countries had prevalence of 10% and above which calls for change of first line regimen as per WHO guidelines on HIVDR [6]. TDR in LICs is more common in NNRTIs (4.5%) and NRTIs (4%) than in protease inhibitors (2.8%) [27] unlike in Europe with less baseline NNRTIs resistance (2.5–2.9%) [31, 32]. Baseline NNRTIs resistance has been shown to cause more impact to treatment response [32]. In LICs, prevalence of TDR in children ≤12 years was found at 42.7% in those exposed to prevention of mother to child transmission and 12.7% in unexposed [23]. These countries now face dilemma of overcoming TDR and this is further complicated by lack of virological monitoring and inaccessible genotyping test. The access to baseline genotyping test in the Strategic Timing of Antiretroviral Treatment trial (START) was found to be 0.1% in Africa, South America (1.8%), and Asia (22%) compared in Europe (86.7%), United States (81.3%) and Australia (89.9%) [25]. Moreover, Sanger sequencing commonly used in these countries cannot detect resistant variants below 20% and yet these variants been associated with treatment failure [33]. Only 22% of patients on cART in middle and LICs get access to virological monitoring [34]. This implies reliance on clinical and immunological monitoring which detect treatment failure late and this leads to emergence of more complicated DRMs. In children below three years, the impact of TDR is even stronger than in adults with odds ratio for failure of 15.3 and has been associated with VF and acquired drug resistance [35].

### Strategies to address challenges associated with current cART and ways to make more potent ART accessible in LICs

The most ART associated challenges in LICs are mainly rooted on HIV drug resistance among other factors like the scarcity of treatment options for HIV infected patients in LICs more so on those failing salvage therapy. Several recommendations can be made on how to address these challenges and improve the lives of people living with HIV in LICs.

Firstly, patent licences should be closely monitored, and local drug manufacturers be encouraged. Drug price is a major factor contributing to lack of access to these drugs especially due to patent restrictions. Third line therapy may be 18 times and seven times more expensive than FL and SL treatment [36]. However, with expired patent licenses and some expiring soon, it’s high time for stake holders to advocate for voluntary and compulsory licensing among other strategies to make these drugs more affordable. Basing on data on active pharmaceutical ingredients exported in and out of India, the cost of treatment of HIV could be as low as USD 90 annually if substantial generic competition is enforced [37].

HIV integrase inhibitor, DTG has shown superior genetic barrier to resistance and potency in patients with DRMs to RAL and elvitegravir. Once daily dosage of DTG can be given to patients initiating cART and those failing RAL-based cART as once or twice daily dosage depending on presence of integrase strand transfer inhibitors (INSTIs) mutations more so Q148K/R/H. National Institutes of Health consultation recommends the use of tenofovir alafenamide fumarate (TAF) which has less bone and kidney toxicity, and DTG, which may help reduce drug resistance and improve adherence in LICs [38].

The compound patent for DTG is expected to expire in 2026 and with licenses on adults and pediatric formulations available to all LICs through Medicines Patent Pool and ViiV Healthcare, market competition of local manufacturers will likely increase access of drug in this setting. The initiative by International drug purchase facility (UNITAID) to enrol DTG in some LICs of Kenya, Uganda and Nigeria [39] should be encouraged.

Neural tube birth defects in children born to mothers who were exposed to DTG during pregnancy still raises concern to the safety of using DTG in pregnant mothers. In a study that compared the birth outcomes between 1729 pregnant women on DTG based ART and 4359 mothers on EFV in Botswana, found no significant differences in the individual outcomes of stillbirth, neonatal death, preterm birth, very preterm birth, small for gestational age, or very small gestational age, and severe side effects between patients on DTG or EFV based ART [40].

In a recent study looking at neural tube defects with DTG treatment in women who started DTG from the time of conception, 426 (0.94%) infants born from mothers who were initiated on DTG at conception, had neural birth defects of encephalocele, myelomeningocele and iniencephaly compared to 11 300 (0.12%) infants born to mothers on non-DTG regimen at conception [41]. In same study, 2812 infants who were born to mothers who initiated DTG during pregnancy none had neural tube defects. These observations suggest DTG associated neural tube defects may be dictated by the time of DTG initiation to pregnant mothers. EFV-based combinations may be the best choice for HIV infected pregnant women initiating ART but with potential reduction in use of NNRTIs based therapy in LICs, more research is necessary in area of DTG use and pregnancy. The current statement of WHO on potential risk of birth defects to infants born of mothers exposed to DTG at a time of inception, is to initiate pregnant women to EFV based regimen which has confirmed efficacy and safety profiles. DTG can only be used in childbearing women only when consistent contraception is guaranteed and where other first line regimens cannot be used [42]. To concur with this statement, a recent study carried across 13 European countries and Thailand investigating association of initiating EFV based ART during conception or first trimester of pregnancy and birth defects, found no significant difference in prevalence of birth defects between EFV based and non-EFV ART based groups [43]. There are also reports linking the use of DTG to abnormal weight gain. In a study assessing weight change in patients switching from EFV/3TC/ emitricabine (FTC) to an INSTI-based regimen, greatest weight gain was observed in patients switched to abacavir/3TC/DTG combination [44]. Another study reviewing patient data from observational SCOLTA project which was looking for drug-related adverse effects in patients who started a regimen containing, DTG, RAL, EVG, DRV or ETR, found no difference in body mass index between patients on INSTIs-based ART and those on non-INSTIs regimens. However, precautions should be taken when drawing conclusions as these were retrospective observational studies. Therefore randomised, controlled studies are still needed to validate these observations.

ETR is largely patented in developing countries and with patent restrictions in leading manufacturing nations of India, China and Brazil, no generic form is available. The patent on novel compound expires in 2026 which is far beyond 2020 target and therefore more advocacies required. DRV has high genetic barrier to resistance and is effective in patients with multidrug resistant viruses [45] and was approved for use in treatment naïve adults in the US and European Union. No high-level resistance to DRV was observed in national survey of 350 patients failing second line therapy in South Africa [13]. At least three DRV associated mutations in combination with multiple protease inhibitor associated mutations are necessary for DRV resistance to occur [46] as shown in POWER 1 and 2 studies. In Madrid study of 1364 genotypes of cART naïve and experienced patients for the impact of HIV subtype to DRV and tipranavir, all 29 non-subtype B cART naïve patients had 100% susceptibility with DRV, and associated DRMs were more common in HIV subtype B virus than in non-subtype B viruses (*P* < 0.001) [47]. In context that majority of patients in LICs fail treatment with multiple DRMs, DRV provides best alternative for those switching to second line therapy. Despite compound patent on DRV expiring in 2013 and patents on pseudopolymorph and/ or on the combination with ritonavir been granted to most LICs, it is still not widely available in LICs. Countries should take advantage of availability of license not to enforce patents on DRV in sub-Saharan Africa and least developed countries to encourage wide manufacturing of generic forms to increase access.

Secondly, access to single pill formulations, drugs with high genetic barrier to resistance, and those with promising results in clinical trials, should be made a priority. Indeed, high TDR prevalence and lack of baseline resistance testing call for robust ART for patients starting on treatment. RPV approved for treatment of NNRTIs-naïve patients is more tolerable and allows simplification due to its single pill formulations of TDF/FTC/RPV, and TDF/TAF/RPV which improves adherence and general response to treatment. ETR is approved for use in NNRTIs experienced patients and has shown high potency to both wild type and NNRTIs resistant virus [48]. However, the high HIVDR due to Y181C after NVP exposure [49] necessitates genotypic test before use of ETR as salvage therapy.

Doravirine being tested in once daily doravirine/3TC/TDF combination, is more tolerable, has high efficacy, and activity against viruses with resistant mutations K103 N and Y181C [50]. It was non-inferior to EFV with 84% vs 81% of patients in doravirine and EFV arms achieving undetectable VL after 48 weeks respectively in phase 3 DRIVE-AHEAD study [51]. Cabotegravir in advanced stages of clinical trials, look promising for its use as long acting injectable with monthly or bimonthly administration for pre-exposure prophylaxis use and treatment of HIV infection. It has shown better safety profile and high acceptability in low risk uninfected participants in on-going clinical trial HPTN 077 [52]. Though there is possibility of cross resistance with already approved INSTIs through Q148 pathway, it still provides better alternative. In addition to minimal side effects, injectable cabotegravir provides convenience, flexibility, and fitness of patient lifestyles which improves adherence. Bictegravir when given in a fixed-dose combination with FTC and TAF, was not inferior to DTG given in DTG/FTC/TAF combination in treatment-naïve patients after 48 weeks in phase 3 of Study 1490 [53]. MK-8591 a long-acting nucleoside reverse transcriptase translocation inhibitor has shown to be a promising long duration treatment and prophylaxis in phase 1b clinical study showing half-life of 2.3 to 56.8 and 78.5 to 128 h with patent and triphosphate form (MK-8591-TP) respectively [54].

Thirdly, HIV drug resistance surveillance should be emphasized as HIV drug resistance is one of indicators of ART program. The impact of HIVDR has been attributed to be 15.6% of AIDS deaths, 9.4% of new infections and 7.9% of ART costs from 2017 to 2021 [55]. Therefore, monitoring of all three forms of HIVDR, TDR, and PDR, and acquired drug resistance, by putting in place checkpoints to track emerging and spreading HIVDR becomes very paramount. This becomes even more crucial at a time when countries are starting all persons infected with HIV on treatment and with increased coverage of prevention of mother to child transmission programs. Much as prevention of mother to child transmission program has drastically reduced the number of babies acquiring HIV from their HIV positive mothers to < 10%, HIVDR especially to NNRTIs has been increasing in children who still become HIV infected through prevention of mother to child transmission program (Table 1). This is mostly attributed to initiating these children to NNRTIs-containing first line treatment due to lack of recommended protease inhibitors in these settings. This therefore calls for more support to ART programs to ensure adequate and reliable supply of child dosage combinations in these countries. It is not uncommon in some of these settings for clinics to provide children formulations to adults by prescribing high dosage where there is shortage of adult regimens. This form of practice affects pediatric HIV treatment programs and may lead to treatment failure in adults.

**Table 1:** Prevalence of HIV pretreatment drug resistance in pediatrics and adults in low-income countries

Type of population	Year	Region	HIV PDR to NNRTIs	Yearly increase in HIV PDR	Reference
a) Children and infants:					
PMTCT exposed	2016	Sub Saharan Africa	32.4% (95% *CI*: 18.7–46.1%)	26.8% between 2004 and 2013	[23]
PMTCT unexposed	2016	Sub Saharan Africa	9.7% (95% *CI*: 4.6–14.8%)	–	[23]
PMTCT exposed	2012–2013	Togo	81.8%	–	[56]
PMTCT exposed	2007–2014	Zambia	–	21.5–40.2% between 2007/2009–2014	[57]
PMTCT exposed	2011–2014	South Africa	54.9%	–	[58]
PMTCT exposed	2011–2014	Mozambique	59.2%	–	[58]
PMTCT exposed	2011–2014	Swaziland	41.2%	–	[58]
PMTCT exposed	2011–2014	Uganda	38.8%	–	[58]
PMTCT exposed	2011–2014	Zimbabwe	74.7%	–	[58]
PMTCT exposed	2010–2013	South Africa	52.0%	–	[59]
PMTCT exposed	2011	South Africa	56.8%	–	[60]
PMTCT unexposed	2010–2011	Uganda	7.5%	–	[61]
b) All					
All	2001–2016	South Africa	11% (7.5–15.9)	23% (16–19)	[5]
All	2001–2016	East Africa	10.1% (5.1–19.4)	17% (5–30)	[5]
All	2001–2016	West and central Africa	7.2% (2.9–16.5)	17% (6–29)	[5]
All	2001–2016	Latin and Caribbean	9.4% (6.6–13.2)	11% (5–18)	[5]
All	2016	South Africa	11%	23%	[6]
All	2016	East Africa	15.5%	29%	[6]
All	2016	West and central Africa	7.2%	17%	[6]
All	2016	Latin and Caribbean	15%	15%	[6]
All	2016	Asia	–	11%	[6]
All	2000–2016	South Africa	8.5%	1.2–fold increase (95% *CI*: 1.13–1.23)	[62]
Type of population		Region	levels of any HIV PDR	Yearly increase in HIV PDR	reference
All	2008–2010	Angola	16%	–	[63]
All	2016	Argentina	18.6%	–	[6]
All	2012–2014	Botswana	10%	–	[64]
All	2003, 2007–2011	Cuba	22%	–	[65]
All	2015	Mexico	15%	–	[66]
All	2013–2014	Papua New Guinea	6%	–	[67]
All	2000–2016	South Africa	10%	1.1–fold increase (95% *CI*: 1.06–1.15)	[62]
All	2004–2014	Global	6.9% in 2010	9% in 2012	[68]

*Abbreviations*: *CI*: Confidence interval, *HIV* Human immune deficiency syndrome, *PDR* Pretreatment drug resistance, *NNRTIs* Non nucleoside reverse transcriptase inhibitors, *PMTCT* Prevention of mothers to child transmission

-: not applicable

Much as the global burden of HIVDR especially in LICs is known and mitigation strategies well stipulated, most of these countries are struggling to implement these strategies due to mostly inadequate resources and lack of political will by governments in some countries. As a result, there is insufficient monitoring of emerging drug resistance. The early warning indicators by WHO for drug resistance provide alternative way of monitoring for emerging drug resistance in this setting. They include offering optimal treatment and according to the guidelines, checking for percentage of patients with loss of follow up after 12 months, looking at percentage of patients retained on ART at 12 months, patients with on time pill pick up/ clinic appointment, drug stock outs, and looking at patients under viral load monitoring and suppression [68]. Despite many challenges in implementing this form of monitoring, it remains the feasible tool to combat the challenge of rising HIVDR in LICs. The HIVDR monitoring programs cannot afford not to prioritise population at most risk; girls and women, men who have sex with other men, sex workers, drug users, and people in fishing communities; who face a lot of stigma and discrimination. Surveillance on access of HIV services to these vulnerable groups needs to be emphasized by ensuring that monitoring and evaluation systems for reporting on implementation are functional. The generic form of DTG has allowed a rapid roll out of this drug in most LICs. It is expected to change the landscape of HIVDR in LICs due to its high genetic barrier to resistance, better tolerability, and safety profiles basing on research commonly done in subtype B viruses. However, more research is still required to access its efficacy in non-B subtypes since DTG associated drug resistance appears to be HIV subtype specific. As such, there should be well articulated treatment guidelines and frequent surveillance of resistance to guide its proper use in ART naïve and highly treatment experienced patients in LICs.

## Conclusions

The availability of more potent ART in LICs is of utmost importance if UNAIDS 2020 and 2030 goals are to be realized and sustained not only in HICs but also in LICs. Countries that have embraced these targets will have to provide treatment to the increasing numbers of newly infected individuals expected to be 800 000 annually from 30 million in 2017 to 36.4 million in 2025 [37]. In countries which rolled out cART earlier, the prevalence of TDR increased by 12.3% in span of four years and will likely keep increasing. With increased drug switches which is associated with TDR, and acquired drug resistance common in LICs, more potent ART offer therapeutic and preventive incentives which will reduce treatment costs and increase drug options. In addition to making these drugs available, governments must address current loopholes through their jurisdictions as well as addressing lack of political commitment and poor policy decisions seen in some countries. It’s also imperative to strengthen quality service delivery in terms of retention of patients to treatment, support for adherence to cART, patient follow up, and adequate drug stocks to help achieve a free AIDS generation.

## Additional file

Additional file 1: Multilingual abstracts in the five official working languages of the United Nations. (PDF 357 kb)

## Data Availability

All data is available in the manuscript.

## Abbreviations

AIDS: Acquired immune deficiency syndrome
ART: Antiretroviral therapy
ATV/r: Atazanavir/ritonavir
cART: Combined antiretroviral therapy
DRMs: Drug resistance mutations
DRV: Darunavir
DRV/r: Darunavir/ritonavir
DTG: Dolutegravir
EFV: Efavirenz
ETR: Etravirine
FL: First line
FTC: Emtricitabine
HICs: High income countries
HIV: Human immune deficiency syndrome
HIVDR: Human immune deficiency drug resistance
INSTIs: Integrase strand transfer inhibitors
LICs: Low-income countries
LPV/r: Lopinavir/ritonavir
NNRTIs: Non nucleoside reverse transcriptase inhibitors
NRTIs: Nucleoside reverse transcriptase inhibitors
NVP: Nevirapine
PDR: Pretreatment drug resistance
RAL: Raltegravir
RPV: Rilpivirine
SL: Second line
TAF: Tenofovir alafenamide fumarate
TDF: Tenofovir
TDR: Transmitted drug resistance
UNAIDS: United Nations programme on HIV and AIDS
UNITAID: International drug purchase facility
WHO: World health organization

## References

[1] UNAIDS (2014). 90-90-90. An ambitious treatment target to help end the AIDS epidemic.

[2] Gourlay A, Noori T, Pharris A, Axelsson M, Costagliola D, Cowan S (2017). The human immunodeficiency virus continuum of Care in European Union Countries in 2013: data and challenges. Clin Infect Dis.

[3] UNAIDS. ENDING AIDS-Progress towards the 90–90-90 targets. http://www.unaids.org/sites/default/files/media_asset/Global_AIDS_update_2017_en.pdf. Accessed 18 Jan 2019.

[4] UNAIDS. Miles to go-closing gaps, breaking barriers, righting injustices. https://www.unaids.org/sites/default/files/media_asset/miles-to-go_en.pdf. Accessed 16 Jan 2019.

[5] Gupta RK, Gregson J, Parkin N, Haile-Selassie H, Tanuri A, Andrade Forero L (2018). HIV-1 drug resistance before initiation or re-initiation of first-line antiretroviral therapy in low-income and middle-income countries: a systematic review and meta-regression analysis. Lancet Infect Dis.

[6] WHO. HIV drug resistance report 2017. http://www.who.int/hiv/topics/drugresistance/en. Accessed 22 Jan 2019.

[7] WHO. Consolidated guidelines on the use of antiretroviral drugs for treating and preventing HIV infection. http://www.who.int/iris/bitstream/10665/85321/1/9789241505727_eng.pdf?ua=1. Accessed 02 Feb 2019.

[8] Boender TS, Hoenderboom BM, Sigaloff KC, Hamers RL, Wellington M, Shamu T (2015). Pretreatment HIV drug resistance increases regimen switches in sub-Saharan Africa. Clin Infect Dis.

[9] WHO. Consolidated guidelines on the use of antiretroviral drugs for treating and preventing HIV infection 2016.http://www.who.int/hiv/pub/arv/arv-2016/en. Accessed 15 Feb 2019.

[10] Phillips AN, Stover J, Cambiano V, Nakagawa F, Jordan MR, Pillay D (2017). Impact of HIV drug resistance on HIV/AIDS-associated mortality, new infections, and antiretroviral therapy program costs in sub–Saharan Africa. J Infect Dis.

[11] Brenner B, Turner D, Oliveira M, Moisi D, Detorio M, Carobene M (2003). A V106M mutation in HIV-1 clade C viruses exposed to efavirenz confers cross-resistance to non-nucleoside reverse transcriptase inhibitors. AIDS..

[12] Ndashimye E, Avino M, Kyeyune F, et al. Absence of HIV-1 Drug Resistance Mutations Supports the Use of Dolutegravir in Uganda. AIDS Res Hum Retroviruses. 2018;34(5):404–14. 10.1089/aid.2017.0205 PMC5934975 29353487

[13] Steegen K, Bronze M, Papathanasopoulos MA, van Zyl G, Goedhals D, Van Vuuren C (2016). Prevalence of antiretroviral drug resistance in patients who are not responding to protease inhibitor-based treatment: results from the first National Survey in South Africa. J Infect Dis.

[14] Rawizza HE, Chaplin B, Meloni ST, Darin KM, Olaitan O, Scarsi KK (2013). Accumulation of protease mutations among patients failing second-line antiretroviral therapy and response to salvage therapy in Nigeria. PLoS One.

[15] TenoRes Study Group (2016). Lancet Infect Dis.

[16] Mannheimer S, Friedland G, Matts J, Child C, Chesney M (2002). The consistency of adherence to antiretroviral therapy predicts biologic outcomes for human immunodeficiency virus-infected persons in clinical trials. Clin Infect Dis.

[17] Garcia de Olalla P, Knobel H, Carmona A, Guelar A, Lopez-Colomes JL, Cayla JA (2002). Impact of adherence and highly active antiretroviral therapy on survival in HIV-infected patients. J Acquir Immune Defic Syndr.

[18] Berg KM, Cooperman NA, Newville H, Arnsten JH (2009). Self-efficacy and depression as mediators of the relationship between pain and antiretroviral adherence. AIDS Care.

[19] Mark D, Armstrong A, Andrade C, Penazzato M, Hatane L, Taing L (2017). HIV treatment and care services for adolescents: a situational analysis of 218 facilities in 23 sub-Saharan African countries. J Int AIDS Soc.

[20] Mills EJ, Lester R, Thorlund K, Lorenzi M, Muldoon K, Kanters S (2014). Interventions to promote adherence to antiretroviral therapy in Africa: a network meta-analysis. Lancet HIV.

[21] Orkin C, DeJesus E, Khanlou H, Stoehr A, Supparatpinyo K, Lathouwers E (2013). Final 192-week efficacy and safety of once-daily darunavir/ritonavir compared with lopinavir/ritonavir in HIV-1-infected treatment-naive patients in the ARTEMIS trial. HIV Med.

[22] LaFleur J, Bress AP, Rosenblatt L, Crook J, Sax PE, Myers J (2017). Cardiovascular outcomes among HIV-infected veterans receiving atazanavir. AIDS..

[23] Boerma RS, Sigaloff KC, Akanmu AS, Inzaule S, Boele van Hensbroek M, Rinke de Wit TF (2017). Alarming increase in pretreatment HIV drug resistance in children living in sub-Saharan Africa: a systematic review and meta-analysis. J Antimicrob Chemother.

[24] Cohen CJ, Andrade-Villanueva J, Clotet B, Fourie J, Johnson MA, Ruxrungtham K (2011). Rilpivirine versus efavirenz with two background nucleoside or nucleotide reverse transcriptase inhibitors in treatment-naive adults infected with HIV-1 (THRIVE): a phase 3, randomised, non-inferiority trial. Lancet..

[25] Baxter JD, Dunn D, White E, Sharma S, Geretti AM, Kozal MJ (2015). Global HIV-1 transmitted drug resistance in the INSIGHT strategic timing of AntiRetroviral treatment (START) trial. HIV Med..

[26] Bansi L, Geretti AM, Dunn D, Hill T, Green H, Fearnhill E (2010). Impact of transmitted drug-resistance on treatment selection and outcome of first-line highly active antiretroviral therapy (HAART). J Acquir Immune Defic Syndr.

[27] Phanuphak P, Sirivichayakul S, Jiamsakul A, Sungkanuparph S, Kumarasamy N, Lee MP (2014). Transmitted drug resistance and antiretroviral treatment outcomes in non-subtype B HIV-1-infected patients in South East Asia. J Acquir Immune Defic Syndr.

[28] Ndembi N, Lyagoba F, Nanteza B, Kushemererwa G, Serwanga J, Katongole-Mbidde E (2008). Transmitted antiretroviral drug resistance surveillance among newly HIV type 1-diagnosed women attending an antenatal clinic in Entebbe. Uganda AIDS Res Hum Retroviruses.

[29] Ndembi N, Hamers RL, Sigaloff KC, Lyagoba F, Magambo B, Nanteza B (2011). Transmitted antiretroviral drug resistance among newly HIV-1 diagnosed young individuals in Kampala. Aids..

[30] Frentz D, Boucher CA, van de Vijver DA (2012). Temporal changes in the epidemiology of transmission of drug-resistant HIV-1 across the world. AIDS Rev.

[31] Derdelinckx I, Van Laethem K, Maes B, Schrooten Y, De Wit S, Florence E (2004). Current levels of drug resistance among therapy-naive HIV-infected patients have significant impact on treatment response. J Acquir Immune Defic Syndr.

[32] Hofstra LM, Sauvageot N, Albert J, Alexiev I, Garcia F, Struck D (2016). Transmission of HIV drug resistance and the predicted effect on current first-line regimens in Europe. Clin Infect Dis.

[33] Kyeyune F, Gibson RM, Nankya I, Venner C, Metha S, Akao J (2016). Low-frequency drug resistance in HIV-infected Ugandans on antiretroviral treatment is associated with regimen failure. Antimicrob Agents Chemother.

[34] WHO. The availability and use of diagnostics for HIV: a 2012/2013 WHO survey of low-and middle-income countries. http://www.who.int/iris/bitstream/10665/147213/1/9789241507905_eng.pdf?ua=1. Accessed 14 Jan 2019.

[35] Kityo C, Boerma RS, Sigaloff KCE, Kaudha E, Calis JCJ, Musiime V (2017). Pretreatment HIV drug resistance results in virological failure and accumulation of additional resistance mutations in Ugandan children. J Antimicrob Chemother.

[36] Médecins Sans Frontières. Untangling the web of antiretroviral price reductions 2016. 18^th^ Edition. https://www.msfaccess.org/untangling-web-antiretroviral-price-reductions-18th-edition. Accessed 13 Jan 2019.

[37] Hill A, et al. Generic treatments for HIV, HBV, HCV, TB could be mass produced for <$90 per patient. 9th international AIDS society conference on HIV science, Paris, abstract TUAD0104, 2017.

[38] Godfrey C, Thigpen MC, Crawford KW, et al. Global HIV Antiretroviral Drug Resistance: A Perspective and Report of a National Institute of Allergy and Infectious Diseases Consultation. J Infect Dis. 2017;216(suppl_9):S798–S800. 10.1093/infdis/jix137 PMC5853929 28973412

[39] UNITAID. Kenya to introduce better treatment for people living with HIV 2017. http://www.unitaid.eu/news-blog/kenya-introduce-better-treatment-people-living-hiv/#en. Accessed 12 Jan 2019.

[40] Zash R, Jacobson DL, Diseko M, Mayondi G, Mmalane M, Essex M (2018). Comparative safety of dolutegravir-based or efavirenz-based antiretroviral treatment started during pregnancy in Botswana: an observational study. Lancet Glob Health.

[41] Zash R, Makhema J, Shapiro RL (2018). Neural-tube defects with Dolutegravir treatment from the time of conception. N Engl J Med.

[42] WHO. Potential safety issue affecting women living with HIV using dolutegravir at the time of conception .https://www.who.int/medicines/publications/drugalerts/Statement_on_DTG_18May_2018final.pdf?ua=1. Accessed 12 Jan 2019.

[43] Martinez de Tejada B (2019). Birth defects after exposure to Efavirenz-based antiretroviral therapy at conception/first trimester of pregnancy: a multicohort analysis. J Acquir Immune Defic Syndr.

[44] Norwood J, Turner M, Bofill C, Rebeiro P, Shepherd B, Bebawy S (2017). Brief report: weight gain in persons with HIV switched from Efavirenz-based to integrase Strand transfer inhibitor-based regimens. J Acquir Immune Defic Syndr.

[45] Fenton C, Perry CM (2007). Darunavir: in the treatment of HIV-1 infection. Drugs..

[46] Clotet B, Bellos N, Molina JM, Cooper D, Goffard JC, Lazzarin A (2007). Efficacy and safety of darunavir-ritonavir at week 48 in treatment-experienced patients with HIV-1 infection in POWER 1 and 2: a pooled subgroup analysis of data from two randomised trials. Lancet..

[47] Poveda E, de Mendoza C, Parkin N, Choe S, Garcia-Gasco P, Corral A (2008). Evidence for different susceptibility to tipranavir and darunavir in patients infected with distinct HIV-1 subtypes. AIDS..

[48] Andries K, Azijn H, Thielemans T, Ludovici D, Kukla M, Heeres J (2004). TMC125, a novel next-generation nonnucleoside reverse transcriptase inhibitor active against nonnucleoside reverse transcriptase inhibitor-resistant human immunodeficiency virus type 1. Antimicrob Agents Chemother.

[49] Llibre JM, Santos JR, Puig T, Molto J, Ruiz L, Paredes R (2008). Prevalence of etravirine-associated mutations in clinical samples with resistance to nevirapine and efavirenz. J Antimicrob Chemother.

[50] Feng M, Sachs NA, Xu M, Grobler J, Blair W, Hazuda DJ (2016). Doravirine suppresses common nonnucleoside reverse transcriptase inhibitor-associated mutants at clinically relevant concentrations. Antimicrob Agents Chemother.

[51] Squires K et al. Fixed dose combination of doravirine/lamivudine/TDF is non-inferior to efavirenz/emitricabine/TDF in treatment-naive adults with HIV-1 infection:week 48 results of the phase 3 DRVE-AHEAD study. 9^th^ international AIDS society conference on HIV science, Paris, abstract TUAB0104LB, 2017.

[52] Randovitz R (2017). Safety, tolerability and pharmacokinetics of long-acting injectable cabotegravir in low-risk HIV-infected women and men: HPTN 077. 9^th^ international AIDS society coneference on HIV science, Paris, abstract TUAC0106LB.

[53] Gallant J (2017). A phase 3 randomised controlled clinical trial of bictegravir in a fixed dose combination, B/F/TAF, vs ABC/DTG/3TC in treatment-naive adults at week 48. 9th international AIDS society conference on HIV science, Paris, abstract MOAB0105LB.

[54] Matthews RP, et al. Single doses as low as 0.5 mg of the novel NRTTI MK-8591 suppress HIV for at least seven days. 9th International AIDS Society Conference on HIV Science, Paris, abstract TUPDB0202LB, 2017.

[55] WHO. Global action plan on HIV drug resistance 2017–2021. https://apps.who.int/iris/bitstream/handle/10665/255883/9789241512848-eng.pdf?sequence=1. Accessed 14 Jan 2019.

[56] Salou M, Butel C, Konou AA, Ekouevi DK, Vidal N, Dossim S (2016). High rates of drug resistance among newly diagnosed HIV-infected children in the National Prevention of mother-to-child transmission program in Togo. Pediatr Infect Dis J.

[57] Poppe LK, Chunda-Liyoka C, Kwon EH, Gondwe C, West JT, Kankasa C (2017). HIV drug resistance in infants increases with changing prevention of mother-to-child transmission regimens. AIDS..

[58] Jordan MR, Penazzato M, Cournil A, Vubil A, Jani I, Hunt G (2017). Human immunodeficiency virus (HIV) drug resistance in African infants and young children newly diagnosed with HIV: a multicountry analysis. Clin Infect Dis.

[59] Kanthula R, Rossouw TM, Feucht UD, van Dyk G, Beck IA, Silverman R (2017). Persistence of HIV drug resistance among south African children given nevirapine to prevent mother-to-child-transmission. AIDS..

[60] Kuhn L, Hunt G, Technau KG, Coovadia A, Ledwaba J, Pickerill S (2014). Drug resistance among newly diagnosed HIV-infected children in the era of more efficacious antiretroviral prophylaxis. AIDS..

[61] Kityo C, Sigaloff KC, Sonia Boender T, Kaudha E, Kayiwa J, Musiime V (2016). HIV drug resistance among children initiating first-line antiretroviral treatment in Uganda. AIDS Res Hum Retrovir.

[62] Chimukangara B, Lessells RJ, Rhee S-Y, Giandhari J, Kharsany ABM, Naidoo K (2019). Trends in pretreatment HIV-1 drug resistance in antiretroviral therapy naive adults in South Africa, 2000-2016: a pooled sequence analysis. Lancet..

[63] Afonso JM, Bello G, Guimaraes ML, Sojka M, Morgado MG (2012). HIV-1 genetic diversity and transmitted drug resistance mutations among patients from the north, central and south regions of Angola. PLoS One.

[64] Rowley CF, MacLeod IJ, Maruapula D, Lekoko B, Gaseitsiwe S, Mine M (2016). Sharp increase in rates of HIV transmitted drug resistance at antenatal clinics in Botswana demonstrates the need for routine surveillance. J Antimicrob Chemother.

[65] Perez L, Kouri V, Aleman Y, Abrahantes Y, Correa C, Aragones C (2013). Antiretroviral drug resistance in HIV-1 therapy-naive patients in Cuba. Infect Genet Evol.

[66] Avila-Rios S, Garcia-Morales C, Matias-Florentino M, Romero-Mora KA, Tapia-Trejo D, Quiroz-Morales VS (2016). Pretreatment HIV-drug resistance in Mexico and its impact on the effectiveness of first-line antiretroviral therapy: a nationally representative 2015 WHO survey. Lancet HIV..

[67] Lavu E, Kave E, Mosoro E, Markby J, Aleksic E, Gare J (2017). High levels of transmitted HIV drug resistance in a study in Papua New Guinea. PLoS One.

[68] WHO. HIV drug resistance surveillance guidance-2015 update. https://apps.who.int/iris/bitstream/handle/10665/204471/9789241510097_eng.pdf?sequence=1. Accessed 2 Jan 2019.

