# Supplementary material for: The urgent need for more potent antiretroviral therapy in low-income countries to achieve UNAIDS 90-90-90 and complete eradication of AIDS by 2030
Source: Infect Dis Poverty. 2019 Aug 2;8:63. doi: 10.1186/s40249-019-0573-1 (PMC6676518; doi:10.1186/s40249-019-0573-1)
Supplement: Supplementary file 1 — Multilingual abstracts in the five official working languages of the United Nations. (PDF 357 kb) [file 40249_2019_573_MOESM1_ESM.pdf]

Translation of the abstract into the five official working languages of the United Nations

الحاجة المُلِحّة إلى مزيد من العلاج القوي المضاد للفيروسات القهقرية في البلدان المنخفضة الدخل لتحقيق برنامج الأمم المتحدة المشترك المعني بفيروس نقص المناعة البشرية / الإيدز 90-90-90 والإبَادَة التامة للإيدز بحلول عام 2030

إيمانويل ندشيمي وإريك ج. آرتس

الملخص

خلفية: أكثر من 90 ٪ من الأفراد المصابين بفيروسُ العَوَزِ المَناعِيِّ البَشَرِيّ (فَيروسُ الإيدز)-1 سيخضعون للعلاج بحلول عام 2020 في إطار الأهداف الدولية لبرنامج الأمم المتحدة المشترك المعني بفيروس الإيدز 90-90-90. وفقًا لمنهج "العلاج للكل" لمنظمة الصحة العالمية هذا العدد سوف يكون حوالي 36.4 مليون شخص في أكثر من 98 ٪ من البلدان المنخفضة الدخل(LICs).

الموضوع الرئيسي : تزداد مقاومة عقاقير ما قبل المعالجة (PDR) إلى حد كبير عن طريق الاستخدام المتكرر للأدوية غير النيوكليوزيدية المضادة لأنزيم المنتسخة المعاكس(NNRTIs)، عقار إيفافيرينز و نيفيرابين زادت مع طرح مضاد للفيروسات القهقرية مجتمعة (CART) مع زيادة سنوية بنسبة 29 ٪ في بعض البلدان المنخفضة الدخل LICs. تجاوزت نسبة مقاومة عقاقير ما قبل المعالجة (PDR) 10% في معظم البلدان منخفضة الدخل LICs والتي تستدعي تغيير نظام الخط الأول إلى فئات أكثر قوة بموجب توصيات منظمة الصحة العالمية. إذا لم يتم تطبيق أي تغيير على الأنظمة في البلدان منخفضة الدخل فمن المقدر أن فيروس نقص المناعة البشرية المكتسب سيشارك في أكثر من 16 ٪ من إجمالي الوفيات ، و 9 ٪ من الإصابات الجديدة ، و 8 ٪ من إجمالي تكاليف العلاج بمضادات الفيروسات القهقرية بحلول عام 2030. لأنها دون المستوى الأمثل، والآثار الجانبية الضارة المرتبطة بأنظمة الأدوية المتاحة حاليا، تشكل جميعها تهديدًا كبيرًا لتحقيق 90 ٪ من مكافحة الفيروس، والقضاء على الإيدز باعتباره تهديدًا للصحة العامة بحلول عام 2030. تتطلب هذه النداءات إدخال سياسات عاجلة تدعو إلى الترخيص الطوعي، والإلزامي للعقاقير الجديدة الأكثر قوة، والتي ينبغي أن تؤكد أيضًا على وصول هذه الأدوية لجميع الأفراد في جميع أنحاء العالم.

الخلاصة: يعتمد تحقيق أهداف برنامج الأمم المتحدة المشترك المعني بفيروس نقص المناعة البشرية / الإيدز لعامي 2020 و 2030 في البلدان المنخفضة الدخل على الحصول على العلاج النشط لمضاد للفيروسات العكوسة مجتمعة (CART) ذي الحاجز الوراثي العالي لمقاومة العقاقير (DR)، والسلامة الأفضل، وبيانات التحمل. من الضروري أيضًا تعزيز تقديم الخدمات عالية الجودة من حيث الاحتفاظ بالمرضى للعلاج، ودعم الالتزام بمضادات الفيروسات القهقرية، ومتابعة المريض، ومخزونات الأدوية المناسبة للمساعدة في تحقيق جيل معافى من الإيدز.

Translated from English version into Arabic by Aalya Al-Beeshi, Revised by Shaima'a al-Fahel, through

TRANSLATORS
WITHOUT BORDERS

要实现联合国艾滋病规划署的 90-90-90 及到 2030 年彻底消除艾滋病目标，低收入国家迫切需要更有效的抗逆转录病毒治疗方案

Emmanuel Ndashimye and Eric J. Arts

摘要

引言：按照联合国艾滋病规划署的 90-90-90 全球目标，到 2020 年，90%以上的 HIV-1 感染者应接受治疗。按照世界卫生组织“全体治疗”的策略，需治疗人数达 3640 万，其中 98%以上的人群生活在低收入国家。

正文：由于频繁使用非核苷逆转录酶抑制剂(NNRTIs)、依法韦伦(efavirenz)和奈韦拉平(nevirapine)，可能会引起预处理药物耐药性(PDR)，而联合抗逆转录病毒治疗(cART)的推进将会进一步增强这些药物的耐药性。在一些低收入国家，这种耐药性年增速为 29%。在大多数低收入国家中，PDR 已超过了 10%。根据世界卫生组织建议，需进一步增强一线治疗方案疗效。若不改进，到 2030 年，HIVDR 造成的死亡病例数将占总数的 16%以上、9%的新发感染和 8%的 cART 总成本。目前可用的药物治疗方案的依从性欠佳，且有副作用，阻碍了在 2030 年如期实现 90%的病毒抑制和消除艾滋病这一公共卫生威胁。在这种情况下，需要紧急制定新政策，提倡自愿及强制为新的更有效的药物颁发药物许可证，同时应该保证全世界所有人都能获得到这些药物。

结论：2020 年和 2030 年联合国艾滋病规划署在低成本战略领域的目标能否实现取决于是否可获得具有更高耐药性遗传屏障、更好的安全性和耐受性的活性 cART。此外，还须提高服务质量，如，保证患者接受治疗，坚持 cART，患者随访和充足的药物，以帮助实现零艾滋病愿景。

Translated from English version into Chinese by Xin-Yu Feng, edited by Jin Chen

CHINESE CENTER FOR DISEASE CONTROL AND PREVENTION
NATIONAL INSTITUTE OF PARASITIC DISEASES

Urgence d’un traitement antirétroviral plus puissant dans les pays à faible revenu pour atteindre les objectifs 90-90-90 de l’ONUSIDA et éradiquer complètement le sida d’ici 2030

Emmanuel Ndashimye et Eric J. Arts

Résumé

Contexte : Plus de 90 % des personnes infectées par le VIH-1 suivront un traitement d’ici 2020 selon les objectifs mondiaux 90-90-90 de l’ONUSIDA. Dans le cadre de l’approche « Traiter tout le monde » de l’OMS, ce nombre sera d’environ 36,4 millions de personnes dont plus de 98 % dans les pays à faible revenu (PFR).

Corps de texte principal : La pharmacorésistance prétraitement (PRPT), principalement causée par l’utilisation fréquente d’inhibiteurs non nucléosidiques de la transcriptase inverse (INNTI), d’efavirenz et de névirapine, a augmenté avec le déploiement du traitement antirétroviral combiné (TARc), une augmentation annuelle de 29 % dans certains pays à faible revenu. La pharmacorésistance prétraitement a dépassé 10 % dans la plupart des pays à faible revenu, ce qui justifie le remplacement du traitement de première intention par des classes de médicaments plus forts, conformes aux recommandations de l’OMS. Si aucun changement dans les protocoles thérapeutiques n’est mis en œuvre dans les pays à faible revenu, on estime que plus de 16 % du nombre total de décès, 9 % des nouvelles infections et 8 % des coûts totaux des TARc seront dus à la pharmacorésistance au VIH d’ici 2030. Le manque d’adhésion et les effets indésirables associés aux protocoles thérapeutiques actuels constituent une menace sérieuse pour l’objectif de 90 % d’élimination du virus et pour l’éradication du sida en tant que menace pour la santé publique

d’ici 2030. Il est donc urgent de mettre en place des politiques préconisant l’octroi volontaire et obligatoire d’autorisations pour de nouveaux médicaments plus puissants et mettant également l’accent sur l’accès universel à ces médicaments pour tous les individus dans le monde.
Conclusions : L’atteinte des objectifs de l’ONUSIDA 2020 et 2030 dans les pays à faible revenu dépend de l’accès à un TARc actif présentant une barrière génétique supérieure à la pharmacorésistance, un plus grand niveau de sécurité et de meilleurs profils de tolérance. Il est également crucial de renforcer la qualité des services fournis en ce qui concerne la rétention des patients au traitement, l’adhésion au TARc, le suivi des patients et le maintien de stocks suffisants de médicaments pour contribuer à atteindre l’objectif d’une génération sans sida.

Translated from English version into French by Eric Cote, Revised by Maëva Paoletti, through

TRANSLATORS
WITHOUT BORDERS

Острая необходимость более эффективной антиретровирусной терапии в странах с низким уровнем дохода для достижения цели ЮНЭЙДС "90-90-90" и полной ликвидации СПИДа к 2030 году.

Эммануэль Ндашимье и Эрик Дж. Артс

Краткое изложение содержания
Справочная информация: Более 90% людей, инфицированных ВИЧ-1, получат лечение к 2020 году в соответствии с глобальными целями ЮНЭЙДС "90-90-90". Согласно подходу ВОЗ «Лечить всех», это число составит примерно 36,4 миллиона человек, более 98% из которых - в странах с низким уровнем дохода (СНД).
Основная часть: Устойчивость к лекарственным препаратам перед лечением (PDR) в значительной степени обусловлена частым использованием ненуклеозидных ингибиторов обратной транскриптазы (ННИОТ), ифавиренца и невирапина, увеличивается с развертыванием комбинированной антиретровирусной терапии (кАРТ) с ежегодным увеличением на 29% в некоторых странах с низким уровнем дохода. PDR превысила 10% в большинстве СНД, что требует изменения лечения препаратами первого ряда на более устойчивые классы в соответствии с рекомендациями ВОЗ. Если никакие изменения в режимах лечения не будут внедрены в СНД, то к 2030 году на долю ВИЧ с устойчивостью к препаратам, по имеющимся оценкам, придется более 16% от общего числа смертей, 9% новых случаев инфицирования и 8% от общих затрат на кАРТ. Неточное соблюдение режима лечения и неблагоприятные побочные эффекты, связанные с доступными в настоящее время схемами приема лекарственных препаратов, - все это представляет серьезную угрозу для достижения к 2030 году подавления вируса на 90% и ликвидации СПИДа как угрозы здоровью населения. Это требует срочного введения политики, которая пропагандирует добровольное и обязательное лицензирование новых более сильнодействующих лекарств,

что должно также придавать особое значение общедоступности этих лекарств для всех людей во всем мире.
Выводы: Достижение целей ЮНЭЙДС на 2020 и 2030 годы в СНД зависит от доступа к активной кАРТ с более высоким генетическим барьером для лекарственной устойчивости (ЛУ), большей безопасности и переносимости лекарственных препаратов. Также необходимо усилить предоставление качественных услуг с точки зрения удержания пациентов на лечении, помощи в соблюдении кАРТ, наблюдения за пациентами и необходимых запасов лекарств, чтобы обеспечить достижение поколения, свободного от СПИДа.

Translated from English version into Russian by Anna Kukharchuk, Revised by Irina Nosova, through

TRANSLATORS
WITHOUT BORDERS

La necesidad urgente de una terapia antirretroviral más potente en los países de bajos ingresos para alcanzar los objetivos 90-90-90 de ONUSIDA y la erradicación definitiva del SIDA para el año 2030

Emmanuel Ndashimye y Eric J. Arts

Resumen
Antecedentes: Más del 90 % de los individuos infectados con VIH-1 estarán bajo tratamiento en el año 2020, según los objetivos globales 90-90-90 de ONUSIDA. Según el enfoque de "tratamiento para todos", esto equivaldría aproximadamente a 36,4 millones de personas, con más del 98 % en los países de bajos ingresos (PBI).
Texto principal: La farmacorresistencia previa al tratamiento (FRP) debida principalmente al uso frecuente de inhibidores no nucleósidos de la transcriptasa inversa (INNTI), efavirenz y nevirapina, ha aumentado con la aplicación de la terapia antirretroviral combinada (TAC), con un aumento anual del 29 % en algunos PBI. La FRP ha superado el 10 % en la mayor parte de los PBI, lo que justifica el cambio de un régimen de primera línea a clases más potentes bajo las recomendaciones de la OMS. Si no se cumplen los cambios en los regímenes de tratamiento en los PBI, se calcula que en el 2030 la farmacorresistencia del VIH contribuirá a más del 16 % de las muertes, 9 % de las nuevas infecciones y 8 % de los costos de las TAC. La adherencia subóptima y los efectos adversos asociados a los regímenes de drogas actualmente disponibles representan una gran amenaza para alcanzar la supresión y eliminación viral del 90 % del SIDA como amenaza para la salud pública para el año 2030. Esto exige introducir en forma urgente políticas que propugnen licencias voluntarias y obligatorias para los fármacos nuevos más potentes, y que además pongan énfasis en el acceso a los mismos para todos los individuos en todo el mundo.
Conclusiones: El logro de los objetivos ONUSIDA 2020 y 2030 en los PBI depende del acceso a una TAC activa, con mayor barrera genética a la resistencia a los fármacos (RF) y mejores perfiles

de tolerancia y seguridad. También es imprescindible fortalecer la prestación de servicios de calidad en términos de permanencia en el tratamiento, apoyo a la adherencia en las TAC, seguimiento de los pacientes y existencias adecuadas de fármacos para contribuir a lograr una generación libre de SIDA.

Translated from English version into Spanish by Beatriz Herrero Migueláñez, Revised by Maria Patricia, through

TRANSLATORS
WITHOUT BORDERS
